# Evaluation of Prognostic Significance of the International Staging System According to Glomerular Filtration Rate in Newly Diagnosed Multiple Myeloma Patients Eligible for Autologous Stem Cell Transplantation

**DOI:** 10.4274/tjh.galenos.2020.2020.0115

**Published:** 2021-02-25

**Authors:** Rafiye Çiftçiler, Hakan Göker, Haluk Demiroğlu, İbrahim Celalettin Haznedaroğlu, Nilgün Sayınalp, Salih Aksu, Osman Özcebe, Yahya Büyükaşık

**Affiliations:** 1 Hacettepe University Faculty of Medicine, Department of Hematology, Ankara, Turkey

**Keywords:** Multiple myeloma, B2-microglobulin, International Staging System

## Abstract

**Objective:**

The prognosis of multiple myeloma (MM) patients is highly heterogeneous. The aim of this study is to determine the impact of patients’ renal functions on the prognostic performance of the International Staging System (ISS). In addition, we aimed to evaluate the results of survival of patients with ISS stages and normal renal functions and those with ISS stages and abnormal renal functions with this study.

**Materials and Methods:**

Two hundred and four patients with newly diagnosed MM who received an autologous stem cell transplantation after induction chemotherapy in our tertiary care center between the years of 2001 and 2018 were evaluated.

**Results:**

There were 153 (75%) MM patients who had a glomerular filtration rate (GFR) of ≥60 mL/min and 51 (25%) MM patients who had GFR of <60 mL/min at the time of diagnosis in this study. There was a strong correlation between ISS stage and GFR. The ISS stages were higher in patients who had GFR of <60 mL/min than patients who had GFR of ≥60 mL/min (p<0.001). Patients with GFR of <60 mL/min were significantly more prevalent in the ISS III group than ISS I and II (p<0.001).

**Conclusion:**

This study showed that the ISS provides significant prognostic information in MM patients with GFR of ≥60 mL/min at diagnosis. However, in patients with impaired renal function at the time of diagnosis, B2-microglobulin may not be a good prognostic indicator since it may be affected by renal dysfunction as well as tumor burden.

## Abstract (tr)

**Amaç:**

Multipl miyelom (MM) hastalarının prognozu oldukça heterojendir. Bu çalışmanın amacı, hastaların böbrek fonksiyonlarının Uluslararası Evreleme Sistemi’nin (ISS) prognostik performansı üzerindeki etkisini belirlemektir. Ayrıca bu çalışma ile ISS evreli ve böbrek fonksiyonları normal ve anormal olan hastaların ISS evresine göre sağkalım sonuçlarını değerlendirmeyi amaçladık.

**Gereç ve Yöntemler:**

2001-2018 yılları arasında üçüncü basamak merkezimizde indüksiyon kemoterapisi sonrası otolog kök hücre nakli yapılan yeni tanı konmuş MM’li 244 hasta değerlendirildi.

**Bulgular:**

Tanı anında glomerüler filtrasyon hızı (GFR) ≥60 mL/dak olan 153 (%75) MM hastası ve GFR’si <60 mL/dak olan 51 (%25) MM hastası vardı. ISS evresi ile GFR arasında güçlü bir ilişki vardı. ISS evreleri, GFR değeri <60 mL/dak olan hastalarda GFR’si ≥60 mL/dak olan hastalara göre daha yüksekti (p<0,001). GFR değeri <60 mL/dak olan hasta sayısı, ISS 3 grubunda ISS 1 ve 2’ye göre anlamlı olarak daha saptandı (p<0,001).

**Sonuç:**

Bu çalışma, ISS’nin GFR’si ≥60 mL/dak olan MM hastalarında tanıda önemli prognostik bilgi sağladığını göstermiştir. Bununla birlikte, tanı anında böbrek fonksiyon bozukluğu olan hastalarda B2-mikroglobulin, böbrek fonksiyon bozukluğundan ve tümör yükünden etkilenebileceğinden iyi bir prognostik gösterge olmayabilir.

## Introduction

The prognosis of multiple myeloma (MM) patients is highly heterogeneous. Tumor characteristics, microenvironment, and host factors such as age, renal insufficiency (RI), and comorbidities have been implicated as signiﬁcant prognostic factors in MM patients. A reproducible and easily assessable prognostic system helps identify patients with poor prognosis and thus helps establish treatment plans for patients with poor prognosis [1]. Greipp et al. [2] developed the International Staging System (ISS) for MM patients. The ISS is based on B2-microglobulin and albumin, which are easily measured and examined in patients. The ISS has been validated and tested in MM patients treated with high-dose melphalan and autologous stem cell transplantation (ASCT) or conventional chemotherapy, and in patients treated with a new course of disease or upfront [3].

RI is one of the major clinical manifestations in MM patients. It is considered as a poor prognostic factor, being associated with shorter survival or earlier death [4]. B2-microglobulin has a strong correlation with tumor burden, but serum B2-microglobulin does not only reﬂect MM. ISS staging increases in patients with elevated B2-microglobulin due to renal failure rather than tumor burden. Since 20%-30% of MM patients present with some degree of RI, the elevation of B2-microglobulin due to renal dysfunction can compromise the prognostic value of the ISS [1]. The aim of this study is to determine the impact of patients’ renal functions on the prognostic performance of the ISS. In addition, we aimed to evaluate the results of survival of patients with ISS stages and normal renal functions and those with ISS stages and abnormal renal functions with this study.

## Materials and Methods

### Study Design, Patients, and Disease Characteristics

This study was performed in a retrospective manner. Demographic data of the patients, ISS staging, and laboratory results were obtained from the hospital database. As a result of the application standards of the hospitals of our tertiary care center, it was recognized from the patient records that all of the studied patients had given informed consent at the time of hospitalization and before the administration of chemotherapy and other relevant diagnostic/therapeutic standards of care. A total of 204 consecutive patients with newly diagnosed MM who underwent ASCT after induction chemotherapy in our tertiary care center between the years of 2001 and 2018 were evaluated. Patients who were not eligible for ASCT were excluded from the study. Patients who received more than one ASCT were also excluded. Patients who received 4-6 courses of induction chemotherapy before ASCT were included in the study. This specific time period was chosen due to the routine use of VCD (bortezomib/cyclophosphamide/dexamethasone), VD (bortezomib/dexamethasone), and VAD (vincristine, doxorubicin and dexamethasone) as induction therapy. Response was determined according to the current International Myeloma Working Group response criteria [5].

### Definitions

Renal function was evaluated with the estimated glomerular ﬁltration rate, which was calculated using the modiﬁed Modiﬁcation of Diet in Renal Disease (MDRD) formula, which uses age, sex, and serum creatinine level (GFR in mL/min/1.73 m^2^) [6]. The degree of RI at the time of diagnosis was staged according to the National Kidney Foundation’s Kidney Disease Outcomes Quality Initiative classiﬁcation of chronic kidney disease (stage 1 with GFR of ≥90 mL/min; stage 2 with GFR of 60-89 mL/min; stage 3 with GFR of 30-59 mL/min; stage 4 with GFR of 15-29 mL/min; and stage 5 with GFR of <15 mL/min or undergoing dialysis) [7].

The ISS classiﬁcation is based on serum albumin and serum B2-microglobulin at the time of initial diagnosis, before the initiation of anti-myeloma therapy [2]. Patients with serum albumin of ≥3.5 g/dL and serum B2-microglobulin of <3.5 mg/L were scored as stage I (ISS I), those with serum B2-microglobulin of ≥5.5 mg/L as stage III (ISS III), and those who did not fulﬁll the stage I or III criteria as stage II (ISS II).

In this study, we formed two groups: one that included patients with at least moderate renal dysfunction, namely patients with GFR of ≥60 mL/min, and another group that included patients with GFR of <60 mL/min.

### Statistical Analysis

Statistical analyses were performed using SPSS 25 (IBM Corp., Armonk, NY, USA). The variables were investigated using visual (histograms, probability plots) and analytical methods (Kolmogorov-Smirnov/Shapiro-Wilk test) to determine whether they were normally distributed or not. Statistical comparisons were made using chi-square tests for categorical data. The Student t-test for two independent samples was used for comparison of continuous numerical data. Survival analyses were performed using the Kaplan-Meier test. Multivariate analysis of predictors of survival was performed using the Cox regression test. Parameters with values of p≤0.20 in univariate tests were included in the multivariate analysis. Values of p<0.05 were considered to indicate statistical significance.

## Results

### Patient Characteristics

A total of 204 patients were enrolled in the study between 2001 and 2018. Patient characteristics are summarized in Table 1. There were 153 (75%) MM patients who had GFR of ≥60 mL/min and 51 (25%) MM patients who had GFR of <60 mL/min at the time of diagnosis. There were 124 (60.8%) males and 80 (39.2%) females with a median age of 58 (range: 35-76) years at the time of diagnosis. The numbers of patients classified with Eastern Cooperative Oncology Group performance status (ECOG PS) 0-1 and 2 were 148 (72.5%) and 56 (27.5%), respectively. There was no statistically significant difference between the two groups in terms of ECOG PS (p=0.27) [8]. No statistically significant difference was found between the groups in terms of age (p=0.36) or sex (p=0.74). There was a strong correlation between ISS stage and GFR. The ISS staging was higher in patients who had GFR of <60 mL/min than patients who had GFR of ≥60 mL/min (p<0.001). The distribution of GFR according to the ISS is depicted in Table 2. Patients with GFR of <60 mL/min were significantly more prevalent in the ISS III group than ISS I and II (p<0.001). Serum hemoglobin levels were higher in patients who had GFR of ≥60 mL/min than patients who had GFR of <60 mL/min (p=0.003). Serum calcium level (p=0.01), B2-microglobulin (p<0.001), and creatinine levels (p<0.001) were statistically significant higher in patients who had GFR of <60 mL/min than patients who had GFR of ≥60 mL/min (p<0.001). Serum platelet counts (p=0.27), lactate dehydrogenase levels (p=0.80), and lytic bone lesions (p=0.08) showed no statistically significant differences between the two groups. There was no statistically significant difference between the two groups in terms of MM types (p=0.64), relapse rates (p=0.82), and mortality rates (p=0.87).

### Overall Outcomes

**Figure 1:**
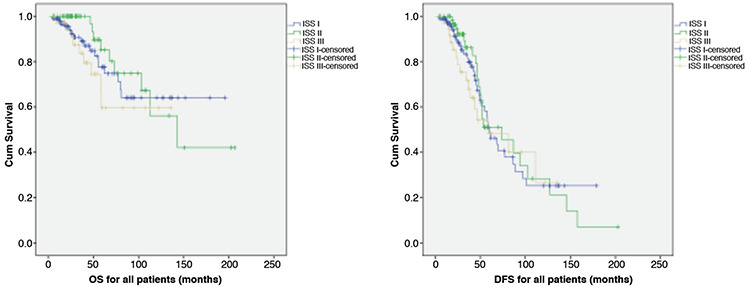
Overall survival (OS) (p=0.36) and disease-free survival (DFS) (p=0.76) for all patients.

The median follow-up period was 35.9 months (range: 4.2-206.4 months) for the entire group. The 5-year overall survival (OS) for all patients was 77% in MM patients staged as ISS I, 85% in MM patients staged as ISS II, and 59% in MM patients staged as ISS III (p=0.36) The 5-year disease-free survival (DFS) rate for all patients was 48% in MM patients staged as ISS I, 51% in MM patients staged as ISS II, and 48% in MM patients staged as ISS III (p=0.76) (Figure 1). 

**Figure 2:**
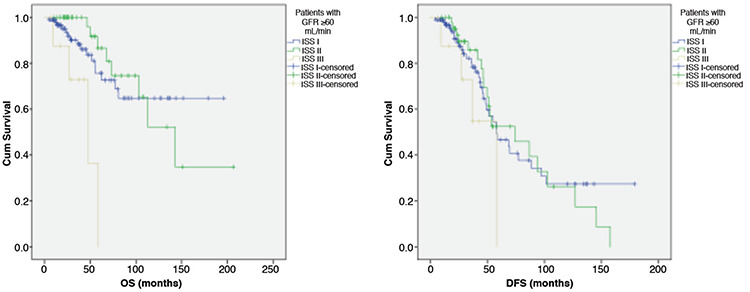
Overall survival (OS) (p=0.002) and disease-free survival (DFS) (p=0.25) for patients with GFR of ≥60 mL/min.

The 5-year OS for patients with GFR of ≥60 mL/min was 75% in MM patients staged as ISS I, 86% in MM patients staged as ISS II, and 0% in MM patients staged as ISS III (p=0.002). The 5-year DFS for patients with GFR of ≥60 mL/min was 46% in MM patients staged as ISS I, 52% in MM patients staged as ISS II, and 0% in MM patients staged as ISS III (p=0.25) (Figure 2).

**Figure 3:**
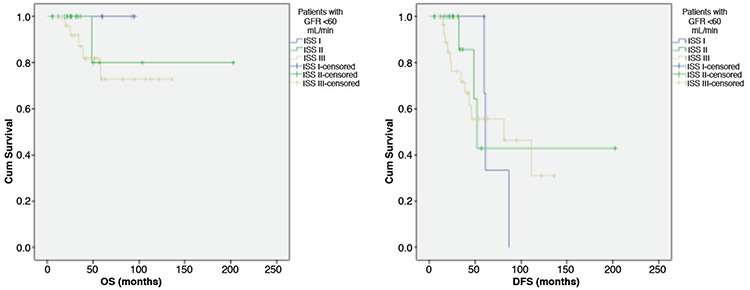
Overall survival (OS) (p=0.46) and disease-free survival (DFS) (p=0.80) for patients with GFR of <60 mL/min.

The 5-year OS for patients with GFR of <60 mL/min was 100% in MM patients staged as ISS I, 80% in MM patients staged as ISS II, and 72% in MM patients staged as ISS III (p=0.46). The 5-year DFS for patients with GFR of <60 mL/min was 66% in MM patients staged as ISS I, 42% in MM patients staged as ISS II, and 55% in MM patients staged as ISS III (p=0.80) (Figure 3).

### Cox Regression Analysis

Univariate and multivariate Cox regression analyses were performed for the factors that could affect OS and DFS. We evaluated Cox regression analysis in two different groups according to the patients’ GFR values. In univariate analyses the factors that affected OS were the age of the patients (p=0.09), ISS staging (ISS I, II) (p=0.17), and ECOG PS (p=0.12) of the patients with GFR of ≥60 mL/min, as shown in Table 3. Cox regression analysis revealed no parameters to predict OS. In univariate analyses, the factors that affected DFS were age of the patients (p=0.09) and ECOG PS (p=0.12) of the patients with GFR of ≥60 mL/min. Cox regression analysis revealed only age of the patients (p=0.009) as being able to predict DFS.

In univariate analyses, the factors that affected OS were lytic bone lesions (p=0.17) and ECOG PS (p=0.02) of the patients with GFR of <60 mL/min, as shown in Table 3. Cox regression analysis revealed ECOG PS and lytic bone lesions to predict OS. In univariate analyses, the factors that affected DFS were lytic bone lesions (p=0.16) and ECOG PS (p=0.01) of the patients with GFR of <60 mL/min. Cox regression analysis revealed only ECOG PS of the patients (p=0.006) as being able to predict DFS (Table 3).

## Discussion

MM is characterized by heterogeneity in the clinical course of the disease and thus risk stratification is essential for prediction of prognosis. For developing a more clinically appropriate, objective, and applicable staging system for MM, the ISS was recommended [2]. Serum B2-microglobulin and serum albumin were evaluated as the most consistent and extensively feasible prognostic factors among a number of statistically significant parameters correlated with survival outcomes [2]. The ISS is based on the measurement of serum albumin and B2-microglobulin levels. However, the cutoff levels have remained a matter of controversy because RI could elevate B2-microglobulin levels even in patients with low tumor burden. Therefore, the ISS cannot consistently provide a good estimate of tumor burden [9]. As a result, it may not accurately predict survival outcomes. In this study, our aim was to evaluate whether the staging of the ISS according to GFR would be effective in predicting the prognosis of patients and survival outcomes.

This study indicated that ISS staging is affected by the degree of GFR. Of the 204 patients we evaluated, GFR was less than 60 mL/min at the time of diagnosis in 51 (25%) patients. The B2-microglobulin levels were significantly higher in these patients than in the patients with GFR of ≥60 mL/min. OS and DFS were not significantly correlated with ISS for all patients. However, when we analyzed patients with GFR of ≥60 mL/min, OS was significantly higher in patients with ISS I and II staging than in patients with ISS III. DFS was not statistically significant in patients with GFR of ≥60, but DFS was higher in patients with ISS I and II staging than in patients with ISS III. When we analyzed the patients with GFR of <60, there was no significant correlation between OS or DFS and ISS stages. The low patient number might have resulted in this lack of a correlation of OS and DFS with ISS. If the number of patients with GFR of <60 mL/min was higher, OS and DFS could have differed statistically. We found that particularly in patients with ISS III disease, which includes the majority of patients with RI, the degree of renal dysfunction as assessed by either GFR or serum creatinine does not have any prognostic impact on OS and DFS in either univariate or multivariate analysis. These results suggest that there may be no prognostic significance of ISS stage in patients with renal failure at the time of diagnosis, because the level of B2-microglobulin due to renal failure will increase even if the tumor burden is low.

The GFR as estimated by the MDRD equation has not been developed to evaluate acute renal failure [6], which is present in many MM patients [10]. A more comprehensive evaluation, including data about renal function before the diagnosis of MM and the course of renal function over a period of time, would be necessary to assess MM-related acute renal failure [1]. However, such data are available only for a small fraction of patients. In this case, it was one of the limitations of our study. Another limitation is that the study was retrospective. Additionally, in this study, the prognostic significance of the ISS was evaluated according to the GFR only in newly diagnosed MM patients eligible for ASCT. Patients who were not eligible for ASCT were excluded from the study.

The incidence of RI in MM varies from series to series. One study showed that 21% of new patients with MM presented with renal failure as defined by serum creatinine of ≥2 mg/dL [11]. Alexanian et al. [12] showed that only 3% of patients with low tumor burden presented with creatinine of >2 mg/dL while 40% of patients with high tumor burden had creatinine of >2 mg/dL. Thus, the study showed that in patients with ISS III disease, which includes many patients with renal failure, elevated B2-microglobulin remains a strong surrogate of tumor burden, despite its increase due to RI [12]. The ISS has been used as an independent prognostic system in recent years, but it is unable to reflect the cytogenetic abnormalities of MM patients. Some new prognostic factors were found using fluorescent in situ hybridization (FISH), karyotypes, and serum free light chains [13,14]. A recent study from the IMWG combined the ISS, calcium, LDH, and FISH data to produce the Revised-ISS (R-ISS) [15]. In another study, the incidence of RI was 31% in newly diagnosed patients with MM, and it was shown to be affected by renal response, treatment, and ISS staging [16]. Renal failure is a prognostic factor in MM patients, as it positively correlates with increased mortality [17]. Goswami et al. [18] have shown that existing MM staging systems (the ISS and Durie-Salmon staging) are not sufficient for distinguishing between risk groups. They reported that certain variables (recurrence after remission, number of regimens used before transplantation, response to induction chemotherapy, serum albumin level, pre-transplant M-protein level) are important factors in predicting OS and DFS. Another recent study showed that pre-transplant induction therapy is an independent prognostic factor for DFS, while ISS stage and post-transplant complete response and very good partial response are independent prognostic factors for OS and DFS [19]. Besides all these, the widespread development, validation, and clinical use of molecular technologies such as FISH and next-generation sequencing have led to the identification of a number of prognostic and predictive biomarkers for DFS, OS, and treatment response [20,21,22].

## Conclusion

This study showed that the ISS provides signiﬁcant prognostic information in MM patients with GFR of ≥60 mL/min at diagnosis. However, in patients with impaired renal function at the time of diagnosis, B2-microglobulin may not be a good prognostic indicator since it may be affected by renal dysfunction as well as tumor burden. The addition of other factors such as LDH, karyotyping, or FISH analysis may improve prognostic ability.

## Figures and Tables

**Table 1:**
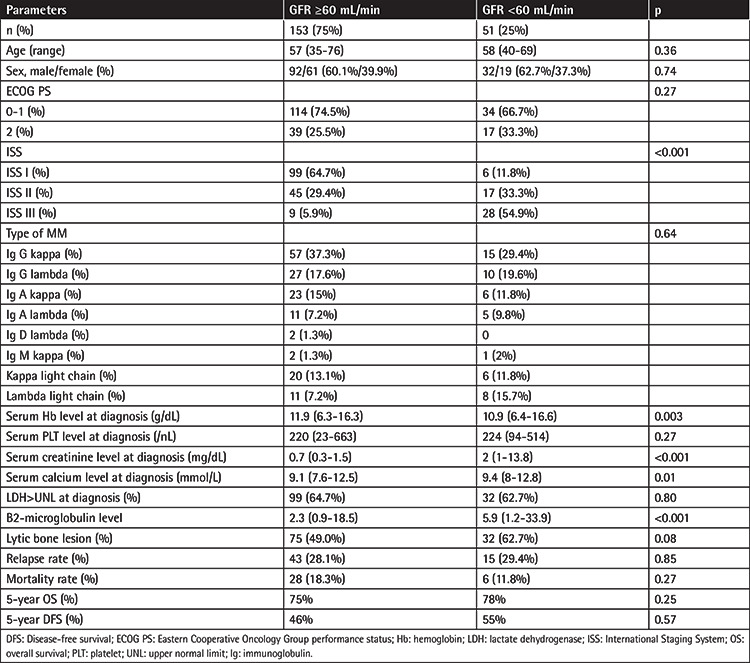
Baseline clinical and demographic characteristics of patients.

**Table 2:**
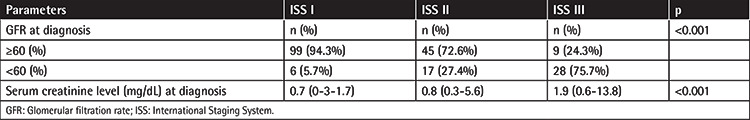
Distribution of patients according to the International Staging System and glomerular filtration rates.

**Table 3:**
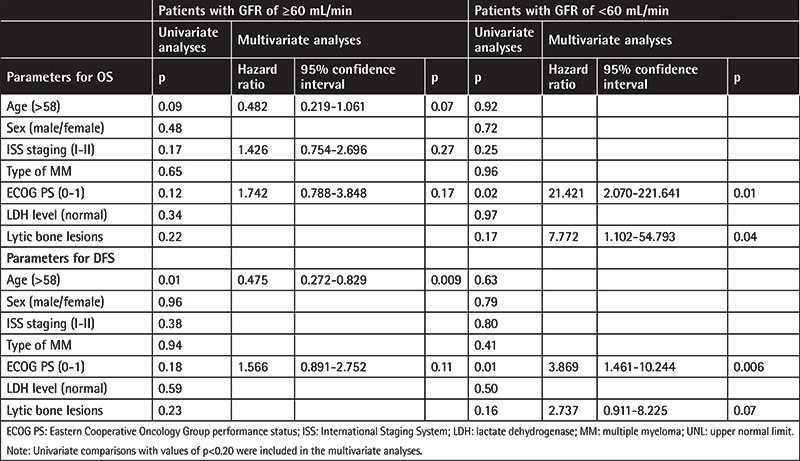
Univariate and multivariate analyses (Cox model) of overall survival and disease-free survival.
